# Chloroform as an Internal Remedy

**Published:** 1866-06

**Authors:** A. P. Merrill

**Affiliations:** New York City


### SELECTIONS.

---

*Chloroform as an Internal Remedy.* By A. P. MERRILL, M. D., New York City.

At the last Commencement of the Bellevue Hospital Medical College, the chosen orator of the occasion, who announced himself as neither an allopath nor a homœopath, neither heroic nor expectant, but only a lawyer, made the startling suggestion, that the three learned Professions are daily lessening their importance, by the liberal and enlightened course of their instructions. As the celebrated teacher, Fenelon, by his skillful instruction of the Dauphin, made his services unnecessary to the King, so do the most skillful theological, legal and medical teachers of the present day, in some degree, supersede the necessity of their future labors, by enlightening mankind in regard to all the practical principles of their several branches of learning. But whatever may be true in regard to theology and law, it cannot be doubted that medical men are slow to believe that the value of their services is ever to be thus lessened, much less dispensed with. Yet there is abundant reason to believe that the tendency of all liberal instruction in medicine is, to inform the masses of mankind in regard to what have been hidden secrets of Professional knowledge, and so to shape the practice of the healing art, as to bring it somewhat largely within the comprehension of the non-professional.

Under the influence of liberal-minded men, anatomy, physiology and chemistry have become common studies of the rising generation in our country, and children in our public schools are permitted to imbibe the facts and principles of natural science in the art of learning to read. Teachers of youth are no longer content with the fictions of nursery tales, and it is by no means uncommon to meet with both men and women, in all the walks of life, who are familiar at once with the nomenclature and leading principles of practical medicine. Nor is the apprehension, now entertained by well-informed Physicians just, that such knowledge will tend to the increase of charlatanry. On the contrary, it is virtually confessed that the better the mysteries and complexities of structure and function in the human system are understood, the less likely will people be to rely upon intuitive skill and superstitious observances, in the treatment of disease. The common sense and common judgment of men, are of scarcely less value in Medicine than in Law and Divinity; and the exclusive and learned charlatanry of each of those learned Professions is to be equally condemned. The single step from the sublime to the ridiculous is not shorter and easier, than that from the complex mysteries of polypharmacy, and the pedantic technicalities of the old school of Medicine, to the simple secrets of the nostrum venders of modern times.

There was a time, not very long ago, when medical prescriptions claimed the confidence of men, by virtue of the multiplicity of their ingredients, and the mysterious characters in which they were written, while the uninitiated were kept in equal ignorance of the nature of the malady and the means of cure; but the introduction of more liberal views, not only simplified all combinations, but by lessening the distance between Physician and patient, induced the latter to expect that his condition will be made known to him, as well as the means provided for relief. The popular knowledge of the most valuable remedies in use is due to the introduction of this more liberal system of medical practice; and all observation proves that men are more likely to require the advice of skillful Physicians than before. As every Doctor, notwithstanding his own knowledge of Medicine, appeals, when attacked by disease, to his neighboring Doctor for advice, so the well-informed non-professional man calls the best skill to his aid, whenever he needs opium, bark or mercury, although he can be taught nothing new as to the remedied qualities of these medicines.

Now, what has become true, under these liberal teachings of the Profession, of bark, opium and mercury, I would have true also of chloroform, as an internal remedy. But in regard to this, we have to contend with preconceived and erroneous notions of its poisonous qualities. These notions, wholly unfounded, have been fostered and encouraged by all classes of people, and have been sanctioned by legislative action, even to the extent of forbidding the sale of any quantity of chloroform, except upon the prescription of a Physician; and when so sold, it must be labelled *poison*, although it is the least poisonous of all the active remedies in use. But we may hope that in the enlightened era the public mind may be disabused of such prejudice, in somewhat less time than it was of a similar prejudice, in regard to Peruvian bark.

Physicians will readily understand something of the value of chloroform when they come to be aware of its power in the chill of fever, and all kindred affections. A remedy which will relieve chill with its concomitant congestions, more or less severe, and that so effectually as to prevent febrile reaction, may be held to possess a power over certain forms of disease, which has been accorded to no other, and to promise results which the most sanguine had never expected to accomplish by medical treatment. The discovery of cinchona and its alkaloids and salts, opened a new era in the practice of the healing art, which is familiar to the human mind throughout the world. The non-professional everywhere have learned the uses of these remedies in the prevention of chill. Everybody is supposed to know that if the life of the patient can be preserved through the chill of fever, and a general reaction established as its natural sequence, there is reason to hope for such a remission of the disease as will justify a reliance upon quinia to prevent its return. This is a great boon to mankind, which could not long remain in obscurity, and the human race has profited by it. But we have a remedy in chloroform more simple, safe and easy of administration, which, striking at the root of the matter, is capable, by a single harmless dose, which any one may administer, of curing the chill itself!

In certain localities where periodic fever prevails in its gravest forms, and especially in subjects with whom the periodic movement has become habitual, it is probable that the disease may not always be eradicated by a single dose of chloroform, although the chill, and the congestions attending upon it, be decidedly relieved. In malarial districts the intensity and persistency of this periodic movement, have often been such as to withstand, for a long while, the most approved antiperiodic treatment, and it is reasonable to expect that under such circumstances preventive measures may become necessary, and even that a return of the chill may demand a repetition of the chloroform treatment. But in several cases in the adult subject, I have found the one hypnotic dose of chloroform sufficient to effect a cure; and I have good reason to believe that in a large majority of cases of infantile convulsions, the single effective dose is all the child requires, although I have often followed it with quinine as a precautionary measure. But in none of these cases has the disease returned, except in one instance, on the thirty-fifth day, whether the quinine was given or not.

It is now nearly fourteen years since I first ventured to pour a teaspoonful of chloroform into the mouth of a child dying of convulsion from chill. The feebleness of the child's constitution, and the long continuance of the convulsion had caused such prostration as to afford little hope of any relief; but the child slept, recovered its pulse and warmth and awoke in apparent health. It had no return of the disease, and from that time to the present I have not failed in any similar case, to accomplish the same result by the same simple means. Sometimes I have succeeded with larger, and sometimes with smaller doses, and in a few cases of great severity, and with the vital powers much exhausted, I have given chloroform by enema, as well as by the mouth, and always continued it until sleep was produced. This being secured, spasmodic action has always ceased, and recuperation begun. Indeed, as soon as the eye-lids are closed the child may be considered safe, and only needs to be permitted to sleep quietly as long as it will.

In a notice of my publication on this subject, a Medical journal remarks that "the convulsions of children are rarely fatal," and in a monograph recently published in this city, it is stated in reference to infantile convulsions, "even in this dreaded disorder the majority will rccover, even if no interference be practiced;" but the mortuary reports of New York, show a frightful amount of mortality for this disease, such as might convince the most inveterate *expectanie*, who ever waited upon Nature to cure a fatal disease, that it is inexpedient to entrust any one case to its remedial agency. Certainly, many of the cases which I have cured with chloroform in hypnotic doses, must have proved fatal without it; having resisted all the usual remedies, and in some instances been abandoned as hopeless.

The following is a brief description of these much dreaded cases of infantile disease: The child is seized, often quite unexpectedly, with all the symptom of chill, as it occurs in the first stage of a febrile paroxysm, but in many instances the chilly sensation is not great or much complained of. In very young children it comes on unnoticed. It does not continue long—sometimes not more than fifteen minutes—before convulsive movements are observed about the mouth and face, extending quickly to the eyes and limbs, until the whole muscular system appears to be involved. The eyelids are stretched wide open, the eye-balls are blood-shot, and twitch with continual spasms, the pupils are dilated, the fingers and toes are drawn in and convulsed, and a quick succession of general convulsions, from which the arteries do not appear to be exempt, continue for one, two, or three hours, during which the pulse becomes enfeebled, the vital powers exausted, the throat and bronchial tubes filled with mucus, and the spasms cease only to be followed by speedy dissolution.

In spite of the remedial powers of nature so strong in the young subject, and in spite of all the artificial remedies heretofore in use, this is the course and the issue of a majority of the cases of infantile convulsions proceeding from the effects of congestivs chill; but my experiments with chloroform prove beyond all question that a hypnotic dose of this remedy given in any part of the course of this alarming and dangerous disease, is certain to afford prompt and permanent relief. The quantity required, depends, as in other diseases and remedies, upon the age and constitution of the patient, and the intensity and duration of the disease; but to obtain its full curative effects, it is always necessary to administer such doses as will produce sleep. This alone is evidence of its constitutional influence, and the rule holds good with new-born infants, and with older children alike.

The remedy is equally efficacious, also, in chills and congestions in the adult subject, in concussion, sunstroke, hemorrhage, cholera morbus, pneumonia, delirium tremens, and in all other diseases dependent upon, or accompanied by severe congestion, as a prominent pathological condition. Indeed, it has in no case failed, in my hands, to relieve congestion, whether proceeding from the febrile cause, local irritation, or concussion. After having observed its wonderful power in cases of infantile convulsions, I have, from analogy alone, been led to use chloroform internally, in a variety of kindred affections, and with uniform success. From the same analogy I infer its efficacy in Asiatic cholera, in the treatment of which, it has, so far as I am informed, not yet been tried in hypnotic doses. In various parts of Europe chloroform has been used in cholera, both by inhalation and in small doses by the stomach, as it has been, in some parts of this country, in congestive chill; but I have not been able to ascertain that in any case reliance has been placed upon its full physiological effects, as evidenced by sleep, which alone insures its curative power. It is not enough that anæthesia is produced by inhalation, or that partial influences over the nervous system in congestive chill, convulsions and cholera are to be overcome, it is only by placing the patient under the full physiological influence of chloroform, through the medium of the stomach and bowels, as evidenced, not by anæsthesia, but by sound and healthful sleep.

The largest quantity of chloroform I have given, was in a case of pulmonary hemorrhage, in which, under urgent circumstances, half a fluid ounce was swallowed in the course of half an hour, with prompt relief to the patient, and without disagreeable or untoward effects. In some cases of infantile convulsion, relieved by full doses of chloroform, the sleep produced, although calm and healthful, was followed by temporary nervous restlessness which subsided without remedies. In pneumonia and delirium tremens, I have given chloroform for several successive days in doses of a fluid drachm and upward, with the invariable effect of producing sleep, and sometimes when large doses of opium had failed, always affording relief to the irritations and congestions of the lungs and brain. In these and other cases, the wakefulness produced by opium has always been relieved by chloroform, and I have sometimes suspected that there was a happy concurrence in the action of the two remedies. In no case have I observed injurious effects upon the mucus tissues, whether the chloroform was administered unmixed or in combination with mucilage or syrup; and my own firm conviction is that we have no other remedy which is powerful for good, that is less objectionable on account of evil results. I do not hesitate to recommend its use in the treatment of cholera, both by the stomach and by enema, depending, as I have done in other cases, upon the force of analogy; but I have little confidence in certain relief, unless the remedy be used in such quantity as may be required in each individual case to produce sleep, or, in other words, its full physiological effects.—*Richmond Medical Journal.*

[With the internal use of chloroform, in the treatment of puerperal convulsions and delirium tremens, we have succeeded when other means failed to give any relief.—Eds.]

